# Impact of type 2 diabetes mellitus on in-hospital-mortality after major cardiovascular events in Spain (2002–2014)

**DOI:** 10.1186/s12933-017-0609-4

**Published:** 2017-10-10

**Authors:** José M. de Miguel-Yanes, Rodrigo Jiménez-García, Valentín Hernández-Barrera, Manuel Méndez-Bailón, Javier de Miguel-Díez, Ana Lopez-de-Andrés

**Affiliations:** 1 Medicine Department, Hospital Universitario Gregorio Marañon, Madrid, Comunidad De Madrid Spain; 2 Preventive Medicine and Public Health Teaching and Research Unit, Health Sciences Faculty, Rey Juan Carlos University, Avda. de Atenas s/n, 28922 Alcorcon, Madrid, Comunidad De Madrid Spain; 3 Medicine Department, Hospital Universitario Clínico San Carlos, Madrid, Comunidad De Madrid Spain; 4 Respiratory Department, Hospital Universitario Gregorio Marañon, Madrid, Comunidad De Madrid Spain

**Keywords:** Type 2 diabetes mellitus, Stroke, Aortic aneurysm and dissection, Acute lower limb ischemia, Acute myocardial infarction, In-hospital mortality

## Abstract

**Background:**

Diabetes mellitus has long been associated with cardiovascular events. Nevertheless, the higher burden of traditional cardiovascular risk factors reported in high-income countries is offset by a more widespread use of preventive measures and revascularization or other invasive procedures. The aim of this investigation is to describe trends in number of cases and outcomes, in-hospital mortality (IHM) and length of hospital stay (LHS), of hospital admissions for major cardiovascular events between type 2 diabetes (T2DM) and matched non-diabetes patients.

**Methods:**

Retrospective study using National Hospital Discharge Database, analyzed in 4 years 2002, 2006, 2010, 2014, in Spain. We included patients (≥ 40 years old) with a primary diagnosis of myocardial infarction, ischemic and hemorrhagic stroke, aortic aneurysm and dissection and acute lower limb ischemia in people with T2DM. Cases were matched with controls (without T2DM) by ICD-9-CM codes, sex, age, province of residence and year.

**Results:**

We selected 130,011 matched couples (50,427 with myocardial infarction, 60,236 with stroke, 2599 with aortic aneurysm and dissection and 16,749 with acute lower limb ischemia. Among T2DM patients we found increasing numbers of admissions overtime for stroke (10,794 in 2002 vs 17,559 in 2014), aortic aneurysm and dissection (390 vs 841) and acute lower limb ischemia (3854 vs. 4548). People were progressively older (except for myocardial infarction), had more comorbidities (especially T2DM patients), and were more frequently coded overtime for cardiovascular risk factors (smoking, obesity, hypertension, lipid disorders) and renal diseases. LHS and IHM declined overtime, though IHM only did it significantly in T2DM patients. Multivariable adjustment showed that T2DM patients had a significantly 15% higher mortality rate during admission for myocardial infarction, a 6% higher mortality for stroke, and a 6% higher mortality rate for “all cardiovascular events combined”, than non-diabetic matched controls.

**Conclusions:**

The number of hospital admissions for stroke, aortic aneurysm and dissection and acute lower limb ischemia increased overtime, but remained stable for myocardial infarction. T2DM is associated to higher IHM after major cardiovascular events. Further research is needed to help us understand the reasons for an apparently increased mortality in T2DM patients when admitted to hospital for some major cardiovascular events.

## Background

Diabetes mellitus is a prevalent chronic condition [1] and has long been associated with cardiovascular events. Nevertheless, the higher burden of traditional cardiovascular risk factors reported in high-income countries is offset by a more widespread use of preventive measures and revascularization or other invasive procedures [2, 3]. This may underlie the fact that in the United States the adjusted incidence rates of hospital admissions for acute myocardial infarction or fatal coronary artery disease have decreased in recent years [4]. We have previously reported increasing incidence rates overtime of acute myocardial infarction [5], ischemic stroke [6] and admissions for abdominal aortic aneurysm [7], but declining rates of major lower extremity amputations due to peripheral artery disease for the overall population [8]. Moreover, incidence rates of major cardiovascular events are higher in people with type 2 diabetes mellitus (T2DM) than among people without diabetes in our Mediterranean population. However, our previous research is based on administrative data used to compare outcomes and procedures between people with or without T2DM who were not matched for baseline characteristics; therefore, some degree of residual confounding cannot be ruled out.

Here we aim to describe trends in number of cases and outcomes, namely in-hospital mortality (IHM) and length of hospital stay (LHS), of hospital admissions for major cardiovascular events (acute myocardial infarction, ischemic and hemorrhagic stroke, aortic aneurysm and dissection and acute lower limb ischemia), analyzed in prespecified moments in time (years 2002, 2006, 2010, 2014), in people with or without T2DM matched for International Classification of Diseases, Ninth Revision, Clinical Modification (ICD-9-CM) codes (up to the fourth digit), sex, age, province of residence and year, using national discharge hospital data.

## Methods

We performed a retrospective, observational study using the Spanish National Hospital Discharge Database (*SNHDD*), which is managed by the Spanish Ministry of Health, Social Services and Equality (MHSSE). The SNHDD includes patient variables (sex and date of birth), admission and discharge dates, up to 14 diagnoses at discharge and up to 20 procedures performed during the hospital stay. The ICD-9-CM is used for coding in the SNHDD.

This study was conducted using the nationwide SNHDD that compiles all public and private hospital data, which covers more than 98% of hospital admissions [9]. The study populations were created as follows.

1. From the entire databases of patients hospitalized in Spain in years 2002, 2006, 2010 and 2014 (data collected between January 1 and December 31) we selected those patients aged 40 years or over who had an ICD9 code for T2DM (codes 250.x0, 250.x2) in any diagnostic position. Patients with type 1 diabetes mellitus were excluded (ICD-9-CM codes 250.x1, 250.x3).
2. Once we had the database with T2DM patients we created five databases for each year including those patients who had in the primary diagnosis of (i) myocardial infarction (codes: 410.xx), (ii) ischemic or hemorrhagic stroke (codes: 431; 432.9; 433.x1; 434.01; 434.11; 434.91), (iii) aortic aneurysm and dissection (codes: 441.xx), (iv) acute lower limb ischemia (codes: 440.21–440.24; 440.4; 444.22; 444.81; 445.02) and (v) any of these four cardiovascular conditions. Therefore we created a total of 20 different study populations of T2DM patients that is, five populations for each year (2002, 2006, 2010 and 2014).
3. Once we had these 20 populations of T2DM patients we matched each T2DM patient with a non-diabetic control using as matching variables ICD-9-CM codes for the four cardiovascular events studied (up to the fourth digit), sex, age, province of residence and year; if more than one control was available for a case, the selection was conducted randomly. The databases used to find the controls were those that included non T2DM patients for years 2002, 2006, 2010 and 2014.
4. So finally we analyzed data from 20 different study populations of T2DM and matched non-diabetic patients. We selected 27,255 matched couples in 2002 with any of the CV events analyzed (12,217 with myocardial infarction, 10,794 with stroke, 390 with aortic aneurysm and dissection, 3854 with acute lower limb ischemia), 32,252 with any of the CV events analyzed in 2006 (13,013 with myocardial infarction, 14,600 with stroke, 615 with aortic aneurysm and dissection and 4024 with acute lower limb ischemia), 35,098 with any of the CV events analyzed in 2010 (12,739 with myocardial infarction, 17,283 with stroke, 753 with aortic aneurysm and dissection and 4323 with acute lower limb ischemia), and 35,406 with any of the CV events analyzed in 2014 (12,458 with myocardial infarction, 17,559 with stroke, 841 with aortic aneurysm and dissection and 4548 with acute lower limb ischemia). We were able to match 80.8% of T2DM cases overall, therefore we analyzed this proportion of all the type 2 diabetic patients that suffered these conditions in Spain in the 4 years studied.

Clinical characteristics included data on overall comorbidities at the time of diagnosis, which were assessed by calculating the Charlson comorbidity index (CCI) [10].

We retrieved data about specific comorbidities, risk factors and therapeutic and diagnostic procedures during the admissions for myocardial infarction, ischemic or hemorrhagic stroke, aortic aneurysm and dissection and acute lower limb ischemia. The conditions and procedures studied and the codes used to identify them according to the ICD-9-CM are shown in Table 1.

**Table 1:** Comorbidities, risk factors and procedures with corresponding ICD-9-CM codes

	ICD-9-CM codes
Current smoking	305.1, V15.82
Obesity	278.xx
Hypertension	401, 401.0, 401.1, 401.9
Lipid metabolism disorders	272.4
Renal disease	403.01, 403.11, 403.91, 404.02, 404.03, 404.12, 404.13, 404.92, 404.93, 582.x, 583.0–583.7, 585.x, 586.x, 588.0, V42.0, V45.1, V56.x
Atrial fibrillation	427.31
Congestive heart failure	398.91, 402.01, 402.11, 402.91, 404.01, 404.03, 404.11, 404.13, 404.91, 404.93, 425.4–425.9, 428.x
Mechanical ventilation	96.7, 96.70, 96.71, 96.72
Hemodyalisis/peritoneal dialysis	39.95, 54.98
Fibrinolysis	99.10
Coronary artery bypass graft (CABG)	36.10–36.19
Percutaneous coronary intervention (PCI)	36.04, 36.06, 36.07, 36.09, 0.66
Decompressive craniectomy	01.24
Carotid endarterectomy	38.11, 38.12
Carotid angioplasty	00.61, 00.62, 00.63, 00.64, 00.65
Thoracic aorta aneurysm open surgery	38.35, 38.45
Endovascular implantation of graft in thoracic aorta (TEVAR)	39.73
Abdominal aorta aneurysm open surgery	38.44
Endovascular implantation of graft in abdominal aorta (EVAR)	39.71
Minor amputation (any lower extremity amputation distal to the ankle joint)	84.10–84.12
Major amputation (any lower extremity amputation through or proximal to the ankle joint)	84.13–84.17
Lower extremity revascularization	39.25, 39.29, 38.38, 38.48
Lower extremity angioplasty	00.60, 39.50

Hospital outcome variables included LHS and IHM, which was defined as the proportion of patients who died during the admission.

### Statistical analysis

A descriptive statistical analysis was performed. Variables were expressed as proportions and means with standard deviations. We constructed bivariate conditional logistic regression models to compare study variables between T2DM patients and controls for each cardiovascular event and for all cardiovascular events combined in the 4 years analyzed.

The changes from year 2002 to 2014 in the means and proportions of study variables were analyzed using ANOVA or Kruskal–Wallis tests for means and logistic regression or a Chi square test for proportions.

To assess the effect of diabetes on the IHM we performed five conditional logistic regression analyses, one for each cardiovascular event and one for “all cardiovascular events combined”. To do so we used diabetes “yes/no” as the dependent variable, and as the independent variables those with significant results in the bivariate analysis and those considered relevant in other studies.

Estimates for variables were expressed as the odds ratios (ORs) with their 95% confidence intervals (CIs).

Cases were matched with controls and all statistical analyses were performed using Stata version 10.1 (Stata, College Station, Texas, USA). Statistical significance was set at p < 0.05 (2-tailed).

### Ethical aspects

Data confidentiality was maintained at all times in accordance with Spanish legislation. Given the anonymous and mandatory nature of the dataset, it was not deemed necessary to obtain informed consent or approval by an ethics committee.

## Results

### Myocardial infarction admissions

The total number of hospitalizations with a primary diagnosis of myocardial infarction analyzed was 100,854. The distribution according to study variables for those with and without T2DM is shown in Table 2. The proportion of men rose from 63.49% in 2002 to 70.79% in 2014 (p < 0.05), and the mean age decreased from 71.01 to 70.23 years (p < 0.05).

**Table 2:** Characteristics, risk factors, and diagnostic and therapeutic procedures for myocardial infarction admissions in type 2 diabetes mellitus (T2DM) versus non-diabetic patients, according to year of study (2002, 2006, 2010, and 2014)

	2002			2006			2010			2014		
	T2DM	No T2DM	p value	T2DM	No T2DM	p value	T2DM	No T2DM	p value	T2DM	No T2DM	p value
N (%)	12,217 (100)	12,217 (100)	NA	13,013 (100)	13,013 (100)	NA	12,739 (100)	12,739 (100)	NA	12,458 (100)	12,458 (100)	NA
Age adjusted rate per 100.000 inhabitants^a^	63.69	NA	NA	61.97	NA	NA	55.86	NA	NA	51.07	NA	NA
Male sex, n (%)^a,b^	7757 (63.49)	7757 (63.49)	NA	8855 (68.05)	8855 (68.05)	NA	8892 (69.8)	8892 (69.8)	NA	8819 (70.79)	8819 (70.79)	NA
Age, mean (SD)^a,b^	71.01 (10.73)	71.01 (10.73)	NA	70.87 (11.49)	70.87 (11.49)	NA	70.5 (12.05)	70.5 (12.05)	NA	70.23 (12.36)	70.23 (12.36)	NA
40–59 years, n (%)	1965 (16.08)	1965 (16.08)	NA	2411 (18.53)	2411 (18.53)	NA	2642 (20.74)	2642 (20.74)	NA	2775 (22.27)	2775 (22.27)	NA
60–69 years, n (%)	2927 (23.96)	2927 (23.96)		2845 (21.86)	2845 (21.86)		2957 (23.21)	2957 (23.21)		3022 (24.26)	3022 (24.26)	
70–79 years, n (%)	4559 (37.32)	4559 (37.32)		4448 (34.18)	4448 (34.18)		3764 (29.55)	3764 (29.55)		3217 (25.82)	3217 (25.82)	
≥ 80 years, n (%)	2766 (22.64)	2766 (22.64)		3309 (25.43)	3309 (25.43)		3376 (26.5)	3376 (26.5)		3444 (27.64)	3444 (27.64)	
CCI, mean (SD)^a,b^	2.65 (0.82)	1.52 (0.74)	< 0.01	2.7 (0.88)	1.56 (0.8)	< 0.01	2.76 (0.9)	1.58 (0.82)	< 0.01	2.8 (0.92)	1.62 (0.85)	< 0.01
Smoking, n (%)^a,b^	2938 (24.05)	3275 (26.81)	< 0.01	4116 (31.63)	4593 (35.3)	< 0.01	4566 (35.84)	5258 (41.27)	< 0.01	4773 (38.31)	5536 (44.44)	< 0.01
Obesity, n (%)^a,b^	1019 (8.34)	565 (4.62)	< 0.01	1488 (11.43)	831 (6.39)	< 0.01	1991 (15.63)	1130 (8.87)	< 0.01	2238 (17.96)	1275 (10.23)	< 0.01
Hypertension, n (%)^a,b^	6047 (49.5)	4687 (38.36)	< 0.01	7511 (57.72)	6038 (46.4)	< 0.01	7576 (59.47)	5954 (46.74)	< 0.01	7401 (59.41)	5938 (47.66)	< 0.01
Lipid metabolism disorders, n (%)^a,b^	1518 (12.43)	1118 (9.15)	< 0.01	2940 (22.59)	2187 (16.81)	< 0.01	4794 (37.63)	3592 (28.2)	< 0.01	5715 (45.87)	4019 (32.26)	< 0.01
Renal diseases, n (%)^a,b^	974 (7.97)	604 (4.94)	< 0.01	1355 (10.41)	853 (6.55)	< 0.01	1755 (13.78)	1031 (8.09)	< 0.01	2034 (16.33)	1287 (10.33)	< 0.01
Atrial fibrillation, n (%)^a,b^	1445 (11.83)	1574 (12.88)	0.011	1592 (12.23)	1707 (13.12)	0.029	1673 (13.13)	1689 (13.26)	0.760	1779 (14.28)	1800 (14.45)	0.697
CHF, n (%)^a^	2842 (23.26)	2263 (18.52)	< 0.01	3125 (24.01)	2357 (18.11)	< 0.01	3175 (24.92)	2307 (18.11)	< 0.01	3171 (25.45)	2331 (18.71)	< 0.01
Mechanical ventilation, n (%)^a^	559 (4.58)	504 (4.13)	0.082	519 (3.99)	484 (3.72)	0.259	445 (3.49)	514 (4.03)	0.022	448 (3.6)	446 (3.58)	0.070
HD/DP, n (%)^a^	83 (0.68)	69 (0.56)	0.257	160 (1.23)	86 (0.66)	< 0.01	172 (1.35)	85 (0.67)	< 0.01	182 (1.46)	95 (0.76)	< 0.01
CABG, n (%)	220 (1.8)	219 (1.79)	0.961	224 (1.72)	210 (1.61)	0.495	246 (1.93)	233 (1.83)	0.547	236 (1.89)	204 (1.64)	0.127
PCI, n (%)^a,b^	2038 (16.68)	2357 (19.29)	< 0.01	4172 (32.06)	4586 (35.24)	< 0.01	5677 (44.56)	6097 (47.86)	< 0.01	6643 (53.32)	7096 (56.96)	< 0.01
Fibrinolysis, n (%)^a,b^	1613 (13.2)	1694 (13.87)	0.109	1289 (9.91)	1451 (11.15)	0.001	906 (7.11)	1048 (8.23)	0.001	463 (3.72)	567 (4.55)	0.001
LHS, mean (SD)^a,b^	10.59 (9.14)	10.08 (9.45)	< 0.01	9.62 (8.7)	8.82 (8.28)	< 0.01	8.47 (9.23)	8.17 (11.51)	0.021	7.58 (7.18)	7.17 (7.86)	< 0.01
In-hospital mortality, n (%)^a,b^	1670 (13.67)	1418 (11.61)	< 0.01	1409 (10.83)	1234 (9.48)	< 0.01	1209 (9.49)	1097 (8.61)	0.011	991 (7.95)	899 (7.22)	0.022

*CCI* charlson comorbidity index, *CHF* congestive heart failure, *HD/DP* hemodyalisis/peritoneal dialysis, *CABG* coronary artery bypass graft, *PCI* percutaneous coronary intervention, *LHS* length of hospital stay

^a^Significant time trend among T2DM sufferers

^b^Significant time trend among non-T2DM sufferers

For all the years analyzed, the mean CCI was higher for T2DM patients when compared with non-diabetic patients.

People with T2DM had higher prevalence of obesity, hypertension and lipid metabolism disorders than non-diabetic people in the 4 years studied, whereas smoking was more frequent among people without diabetes. Overtime the prevalence of these four cardiovascular risk factors increased significantly in both groups.

The proportion of diabetic patients hospitalized with a myocardial infarction who suffered concomitant renal disease or congestive heart failure was higher than for non-diabetic patients.

Regarding the use of therapeutic procedures, among T2DM patients percutaneous coronary intervention (PCI) was recorded in 16.68% in year 2002 and in 53.32% in year 2014 (p < 0.05). However, the use of PCI was lower among diabetic than among matched non-diabetic controls in all the years analyzed. The proportion of patients undergoing coronary artery by-pass grafting (CABG) was similar in both groups and remained stable overtime.

The LHS was longer among T2DM patients than among non-diabetic patients, and decreased significantly from 2002 to 2014.

The IHM for the diabetic sample was 13.67% in 2002 and 7.95% in 2014 (p < 0.05). Equivalent figures for non-diabetic patients were significantly lower, 11.61 and 7.22% respectively.

### Stroke admissions

Table 3 shows the characteristics, risk factors, and diagnostic and therapeutic procedures for stroke admissions in T2DM versus non-diabetic patients, according to the year of study (2002, 2006, 2010, and 2014). The number of T2DM patients and matched controls rose from 10,794 in 2002 to 17,559 in 2014.

**Table 3:** Characteristics, risk factors, and diagnostic and therapeutic procedures for stroke admissions in type 2 diabetes mellitus (T2DM) versus non-diabetic patients, according to year of study (2002, 2006, 2010, and 2014)

	2002			2006			2010			2014		
	T2DM	No T2DM	p value	T2DM	No T2DM	p value	T2DM	No T2DM	p value	T2DM	No T2DM	p value
N (%)^a,b^	10,794 (100)	10,794 (100)	NA	14,600 (100)	14,600 (100)	NA	17,283 (100)	17,283 (100)	NA	17,559 (100)	17,559 (100)	NA
Age adjusted rate per 100.000 inhabitants^a^	56.27	NA	NA	69.53	NA	NA	75.79	NA	NA	71.98	NA	NA
Male sex, n (%)	5706 (52.86)	5706 (52.86)	NA	7795 (53.39)	7795 (53.39)	NA	9345 (54.07)	9345 (54.07)	NA	9581 (54.56)	9581 (54.56)	NA
Age, mean (SD)^a,b^	73.17 (9.75)	73.17 (9.75)	NA	74.15 (10.3)	74.15 (10.3)	NA	74.84 (10.66)	74.84 (10.66)	NA	75.35 (11.06)	75.35 (11.06)	NA
40–59 years, n (%)	1071 (9.92)	1071 (9.92)	NA	1467 (10.05)	1467 (10.05)	NA	1743 (10.09)	1743 (10.09)	NA	1865 (10.62)	1865 (10.62)	NA
60–69 years, n (%)	2386 (22.1)	2386 (22.1)		2695 (18.46)	2695 (18.46)		3027 (17.51)	3027 (17.51)		3046 (17.35)	3046 (17.35)	
70–79 years, n (%)	4436 (41.1)	4436 (41.1)		5593 (38.31)	5593 (38.31)		5923 (34.27)	5923 (34.27)		5224 (29.75)	5224 (29.75)	
≥ 80 years, n (%)	2901 (26.88)	2901 (26.88)		4845 (33.18)	4845 (33.18)		6590 (38.13)	6590 (38.13)		7424 (42.28)	7424 (42.28)	
CCI, mean (SD)^a,b^	2.55 (0.74)	1.51 (0.71)	< 0.01	2.64 (0.79)	1.56 (0.75)	< 0.01	2.69 (0.81)	1.62 (0.78)	< 0.01	2.86 (0.87)	1.8 (0.84)	< 0.01
Smoking, n (%)^a,b^	1414 (13.1)	1566 (14.51)	0.001	2591 (17.75)	2988 (20.47)	< 0.01	3440 (19.9)	3675 (21.26)	< 0.01	3719 (21.18)	4053 (23.08)	< 0.01
Obesity, n (%)^a,b^	617 (5.72)	339 (3.14)	< 0.01	1171 (8.02)	622 (4.26)	< 0.01	1638 (9.48)	873 (5.05)	< 0.01	1985 (11.3)	1056 (6.01)	< 0.01
Hypertension, n (%)^a,b^	6470 (59.94)	5309 (49.18)	< 0.01	9841 (67.4)	7942 (54.4)	< 0.01	11,799 (68.27)	9688 (56.06)	< 0.01	11,818 (67.3)	9804 (55.83)	< 0.01
Lipid metabolism disorders, n (%)^a,b^	770 (7.13)	488 (4.52)	< 0.01	2449 (16.77)	1614 (11.05)	< 0.01	4960 (28.7)	3402 (19.68)	< 0.01	6801 (38.73)	4481 (25.52)	< 0.01
Renal diseases, n (%)^a,b^	511 (4.73)	376 (3.48)	< 0.01	988 (6.77)	625 (4.28)	< 0.01	1634 (9.45)	1009 (5.84)	< 0.01	2233 (12.72)	1410 (8.03)	< 0.01
Atrial fibrillation, n (%)^a,b^	2045 (18.95)	2275 (21.08)	< 0.01	3242 (22.21)	3360 (23.01)	0.065	4222 (24.43)	4395 (25.43)	0.016	4698 (26.76)	4771 (27.17)	0.314
CHF, n (%)^a,b^	503 (4.66)	486 (4.5)	0.579	902 (6.18)	707 (4.84)	< 0.01	1211 (7.01)	1065 (6.16)	0.001	1291 (7.35)	1039 (5.92)	< 0.01
Mechanical ventilation, n (%)^a,b^	254 (2.35)	358 (3.32)	< 0.01	404 (2.77)	472 (3.23)	0.013	523 (3.03)	604 (3.49)	0.010	567 (3.23)	727 (4.14)	< 0.01
HD/DP, n (%)^a^	32 (0.3)	18 (0.17)	0.051	58 (0.4)	31 (0.21)	0.004	73 (0.42)	51 (0.3)	0.049	99 (0.56)	47 (0.27)	< 0.01
Fibrinolysis, n (%)^a,b^	15 (0.14)	22 (0.2)	0.253	109 (0.75)	221 (1.51)	0.000	569 (3.29)	880 (5.09)	< 0.01	812 (4.62)	1220 (6.95)	< 0.01
Decompressive craniectomy, n (%)^b^	31 (0.29)	31 (0.29)	0.502	29 (0.2)	35 (0.24)	0.454	47 (0.27)	70 (0.41)	0.035	53 (0.3)	81 (0.46)	0.015
Carotid endarterectomy, n (%)^a^	31 (0.29)	41 (0.38)	0.227	33 (0.23)	50 (0.34)	0.064	82 (0.47)	62 (0.36)	0.088	85 (0.48)	80 (0.46)	0.694
Carotid angioplasty, n (%)^a,b^	0 (0)	0 (0)	NA	29 (0.2)	24 (0.16)	0.485	68 (0.39)	101 (0.58)	0.011	125 (0.71)	150 (0.85)	0.125
LHS, mean (SD)^a,b^	12.9 (15.1)	12.65 (16.45)	0.254	12.14 (14.63)	11.92 (15.54)	0.204	11.22 (14.56)	11.22 (15.87)	0.977	9.82 (11.75)	10.02 (12.18)	0.119
In-hospital mortality, n (%)^a,b^	1616 (14.97)	1692 (15.68)	0.135	2261 (15.49)	2258 (15.47)	0.960	2582 (14.94)	2568 (14.86)	0.826	2506 (14.27)	2438 (13.88)	0.272

*CCI* charlson comorbidity index, *CHF* congestive heart failure, *HD/DP* hemodyalisis/peritoneal dialysis, *LHS* length of hospital stay

^a^Significant time trend among T2DM sufferers

^b^Significant time trend among non-T2DM sufferers

Men represented around 52–54% of the study sample and the mean age increased from 73.17 years in 2002 to 75.35 years in 2014.

As can be seen in Table 3 the differences in the prevalences between those with and without diabetes, and in the time trends observed for cardiovascular risk factors and concomitant chronic conditions were very similar to those found for myocardial infarction (Table 2).

T2DM patients less frequently received mechanical ventilation and fibrinolysis than matched controls, yet the use of these therapeutic procedures increased overtime.

The LHS and the IHM did not differ significantly between both groups of patients in any year analyzed. However the LHS decreased significantly from around 12 to 10 days from 2002 to 2014. The reduction in the IHM was from 14.97% in year 2002 to 14.27% in year 2014 among T2DM patients (p < 0.05).

### Aortic aneurysm and dissection admissions

As can be seen in Table 4 the number of T2DM patients hospitalized with aortic aneurysm and dissection increased from 390 in 2002 to 841 in 2014. Most of these patients were male (> 93%) and with a mean age ranging from 69 to 72 years.

**Table 4:** Characteristics, risk factors, and diagnostic and therapeutic procedures for aortic aneurysm and dissection admissions in type 2 diabetes mellitus (T2DM) versus non-diabetic patients, according to year of study (2002, 2006, 2010, and 2014)

	2002			2006			2010			2014		
	T2DM	No T2DM	p value	T2DM	No T2DM	p value	T2DM	No T2DM	p value	T2DM	No T2DM	p value
N (%)^a,b^	390 (100)	390 (100)	NA	615 (100)	615 (100)	NA	753 (100)	753 (100)	NA	841 (100)	841 (100)	NA
Age adjusted rate per 100.000 inhabitants	2.03	NA	NA	2.93	NA	NA	3.3	NA	NA	3.45	NA	NA
Male sex, n (%)	364 (93.33)	364 (93.33)	NA	591 (96.1)	591 (96.1)	NA	710 (94.29)	710 (94.29)	NA	802 (95.36)	802 (95.36)	NA
Age, mean (SD)^a,b^	69.84 (9.03)	69.84 (9.03)	NA	71.06 (8.12)	71.06 (8.12)	NA	71.43 (8.08)	71.43 (8.08)	NA	72.41 (7.85)	72.41 (7.85)	NA
40–59 years, n (%)	56 (14.36)	56 (14.36)	NA	60 (9.76)	60 (9.76)	NA	55 (7.3)	55 (7.3)	NA	51 (6.06)	51 (6.06)	NA
60–69 years, n (%)	106 (27.18)	106 (27.18)		177 (28.78)	177 (28.78)		234 (31.08)	234 (31.08)		247 (29.37)	247 (29.37)	
70–79 years, n (%)	184 (47.18)	184 (47.18)		288 (46.83)	288 (46.83)		350 (46.48)	350 (46.48)		376 (44.71)	376 (44.71)	
≥ 80 years, n (%)	44 (11.28)	44 (11.28)		90 (14.63)	90 (14.63)		114 (15.14)	114 (15.14)		167 (19.86)	167 (19.86)	
CCI, mean (SD)^a,b^	2.48 (0.82)	1.42 (0.82)	< 0.01	2.56 (0.84)	1.49 (0.79)	< 0.01	2.59 (0.76)	1.53 (0.79)	< 0.01	2.7 (0.84)	1.59 (0.75)	< 0.01
Smoking, n (%)^a,b^	139 (35.64)	136 (34.87)	0.802	238 (38.7)	214 (34.8)	0.145	323 (42.9)	316 (41.97)	0.702	363 (43.16)	360 (42.81)	0.879
Obesity, n (%)^a,b^	19 (4.87)	11 (2.82)	0.149	32 (5.2)	20 (3.25)	0.099	79 (10.49)	28 (3.72)	< 0.01	82 (9.75)	45 (5.35)	0.001
Hypertension, n (%)^a,b^	213 (54.62)	139 (35.64)	< 0.01	367 (59.67)	269 (43.74)	< 0.01	470 (62.42)	371 (49.27)	< 0.01	541 (64.33)	430 (51.13)	< 0.01
Lipid metabolism disorders, n (%)^a,b^	47 (12.05)	20 (5.13)	0.001	129 (20.98)	83 (13.5)	0.001	292 (38.78)	200 (26.56)	< 0.01	427 (50.77)	292 (34.72)	< 0.01
Renal diseases, n (%)^a,b^	31 (7.95)	28 (7.18)	0.686	67 (10.89)	62 (10.08)	0.635	114 (15.14)	84 (11.16)	0.021	147 (17.48)	118 (14.03)	0.053
Atrial fibrillation, n (%)^a,b^	38 (9.74)	26 (6.67)	0.112	58 (9.43)	67 (10.89)	0.376	90 (11.95)	106 (14.08)	0.212	157 (18.67)	116 (13.79)	0.007
CHF, n (%)	22 (5.64)	13 (3.33)	0.100	29 (4.72)	24 (3.9)	0.493	44 (5.84)	40 (5.31)	0.638	41 (4.88)	34 (4.04)	0.407
Mechanical ventilation, n (%)	36 (9.23)	32 (8.21)	0.593	55 (8.94)	48 (7.8)	0.483	36 (4.78)	60 (7.97)	0.009	61 (7.25)	62 (7.37)	0.919
HD/DP, n (%)	7 (1.79)	5 (1.28)	0.566	18 (2.93)	14 (2.28)	0.467	16 (2.12)	23 (3.05)	0.240	25 (2.97)	24 (2.85)	0.884
Thoracic aorta aneurysm open surgery, n (%)^a,b^	9 (2.31)	9 (2.31)	0.903	22 (3.58)	30 (4.88)	0.198	25 (3.32)	46 (6.11)	0.005	43 (5.11)	50 (5.95)	0.379
TEVAR, n (%)	0 (0)	0 (0)	NA	0 (0)	0 (0)	NA	25 (3.32)	16 (2.12)	0.133	37 (4.4)	32 (3.8)	0.508
Abdominal aorta aneurysm open surgery, n (%)^a,b^	86 (22.05)	94 (24.1)	0.454	111 (18.05)	112 (18.21)	0.940	100 (13.28)	128 (17)	0.038	137 (16.29)	153 (18.19)	0.281
EVAR, n (%)^a,b^	19 (4.87)	22 (5.64)	0.640	93 (15.12)	76 (12.36)	0.139	232 (30.81)	190 (25.23)	0.013	312 (37.1)	291 (34.6)	0.250
LHS, mean (SD)^a,b^	15.63 (16.07)	12.88 (12.95)	0.008	11.82 (12.11)	11.44 (16.71)	0.644	11.28 (18.3)	10.92 (13.32)	0.669	9.43 (13.33)	9.63 (13.24)	0.760
In-hospital mortality, n (%)^a^	56 (14.36)	50 (12.82)	0.447	77 (12.52)	78 (12.68)	0.920	82 (10.89)	90 (11.95)	0.446	68 (8.09)	84 (9.99)	0.118

*CCI* charlson comorbidity index, *CHF* congestive heart failure, *HD/DP* hemodyalisis/peritoneal dialysis, *TEVAR* endovascular implantation of graft in thoracic aorta, *EVAR* endovascular implantation of graft in abdominal aorta, *LHS* length of hospital stay

^a^Significant time trend among T2DM sufferers

^b^Significant time trend among non-T2DM sufferers

The prevalence of obesity, hypertension and lipid metabolism disorders were higher among T2DM patients in the 4 years analyzed and increased significantly overtime in both groups.

No differences were found in the use of open or endovascular procedures to treat thoracic or abdominal aortic aneurysms between T2DM and non-diabetic patients. However a relevant and significant increment was observed for the use of endovascular procedures, especially for abdominal aneurysm, which increased from 4.87 to 37.1% over the study period among T2DM patients.

LHS and IHM were similar in both groups. The LHS decreased significantly from around 16–13 days from 2002 to 2014 in the T2DM group, but it increased slightly yet significantly in the non-T2DM group. Overtime IHM decreased significantly only in the T2DM group (14.36% in 2002 vs. 8.09% in 2014).

### Acute lower limb ischemia admissions

The number of T2DM patients hospitalized with an acute lower limb ischemia increased from 3854 in 2002 to 4548 in 2014, as can been seen in Table 5. Males represented around 73% and the mean age was close to 74 years.

**Table 5:** Characteristics, risk factors, and diagnostic and therapeutic procedures for acute lower limb ischemia admissions in type 2 diabetes mellitus (T2DM) versus non-diabetic patients, according to year of study (2002, 2006, 2010, and 2014)

	2002			2006			2010			2014		
	T2DM	No T2DM	p value	T2DM	No T2DM	p value	T2DM	No T2DM	p value	T2DM	No T2DM	p value
N (%)^a,b^	3854 (100)	3854 (100)	NA	4024 (100)	4024 (100)	NA	4323 (100)	4323 (100)	NA	4548 (100)	4548 (100)	NA
Age adjusted rate per 100.000 inhabitants^a^	20.09	NA	NA	19.16	NA	NA	18.96	NA	NA	18.64	NA	NA
Male sex, n (%)	2850 (73.95)	2850 (73.95)	NA	3024 (75.15)	3024 (75.15)	NA	3221 (74.51)	3221 (74.51)	NA	3356 (73.79)	3356 (73.79)	NA
Age, mean (SD)^a,b^	72.27 (10.54)	72.27 (10.54)	NA	72.49 (10.94)	72.49 (10.94)	NA	72.69 (11.69)	72.69 (11.69)	NA	73.11 (11.84)	73.11 (11.84)	NA
40–59 years, n (%)	524 (13.6)	524 (13.6)	NA	565 (14.04)	565 (14.04)	NA	697 (16.12)	697 (16.12)	NA	690 (15.17)	690 (15.17)	NA
60–69 years, n (%)	851 (22.08)	851 (22.08)		890 (22.12)	890 (22.12)		935 (21.63)	935 (21.63)		1044 (22.96)	1044 (22.96)	
70–79 years, n (%)	1470 (38.14)	1470 (38.14)		1409 (35.01)	1409 (35.01)		1291 (29.86)	1291 (29.86)		1205 (26.5)	1205 (26.5)	
≥ 80 years, n (%)	1009 (26.18)	1009 (26.18)		1160 (28.83)	1160 (28.83)		1400 (32.38)	1400 (32.38)		1609 (35.38)	1609 (35.38)	
CCI, mean (SD)^a,b^	2.33 (0.82)	1.34 (0.83)	< 0.01	2.42 (0.85)	1.38 (0.89)	< 0.01	2.46 (0.9)	1.43 (0.92)	< 0.01	2.51 (0.86)	1.49 (0.89)	< 0.01
Smoking, n (%)^a,b^	902 (23.4)	1037 (26.91)	< 0.01	1156 (28.73)	1334 (33.15)	< 0.01	1439 (33.29)	1645 (38.05)	0.000	1625 (35.73)	1908 (41.95)	< 0.01
Obesity, n (%)^a,b^	82 (2.13)	49 (1.27)	0.004	161 (4)	79 (1.96)	< 0.01	225 (5.2)	126 (2.91)	< 0.01	346 (7.61)	175 (3.85)	< 0.01
Hypertension, n (%)^a,b^	1719 (44.6)	1293 (33.55)	< 0.01	2286 (56.81)	1595 (39.64)	< 0.01	2626 (60.74)	1950 (45.11)	< 0.01	2763 (60.75)	2172 (47.76)	< 0.01
Lipid metabolism disorders, n (%)^a,b^	239 (6.2)	114 (2.96)	< 0.01	658 (16.35)	354 (8.8)	< 0.01	1415 (32.73)	818 (18.92)	< 0.01	1875 (41.23)	1218 (26.78)	< 0.01
Renal diseases, n (%)^a,b^	356 (9.24)	264 (6.85)	< 0.01	514 (12.77)	359 (8.92)	< 0.01	747 (17.28)	514 (11.89)	< 0.01	870 (19.13)	612 (13.46)	< 0.01
Atrial fibrillation, n (%)^a,b^	474 (12.3)	589 (15.28)	< 0.01	591 (14.69)	615 (15.28)	0.417	722 (16.7)	768 (17.77)	0.160	792 (17.41)	901 (19.81)	0.002
CHF, n (%)^a,b^	238 (6.18)	223 (5.79)	0.467	291 (7.23)	235 (5.84)	0.011	362 (8.37)	351 (8.12)	0.662	368 (8.09)	355 (7.81)	0.610
Mechanical ventilation, n (%)	23 (0.6)	29 (0.75)	0.406	36 (0.89)	57 (1.42)	0.029	21 (0.49)	49 (1.13)	0.001	29 (0.64)	35 (0.77)	0.447
HD/DP, n (%)^a,b^	59 (1.53)	48 (1.25)	0.288	50 (1.24)	59 (1.47)	0.389	67 (1.55)	85 (1.97)	0.135	93 (2.04)	83 (1.82)	0.443
Minor amputation, n (%)	577 (14.97)	353 (9.16)	< 0.01	620 (15.41)	333 (8.28)	< 0.01	633 (14.64)	341 (7.89)	< 0.01	645 (14.18)	382 (8.4)	< 0.01
Major amputation, n (%)^a,b^	584 (15.15)	621 (16.11)	0.217	611 (15.18)	596 (14.81)	0.608	558 (12.91)	538 (12.45)	0.494	489 (10.75)	537 (11.81)	0.090
Lower extremity revascularization, n (%)^a,b^	910 (23.61)	856 (22.21)	0.130	933 (23.19)	894 (22.22)	0.301	825 (19.08)	900 (20.82)	0.038	749 (16.47)	892 (19.61)	< 0.01
Lower extremity angioplasty, n (%)^a,b^	347 (9)	319 (8.28)	0.247	570 (14.17)	473 (11.75)	0.001	1034 (23.92)	814 (18.83)	< 0.01	1415 (31.11)	1103 (24.25)	< 0.01
LHS, mean (SD)^a,b^	16.48 (16.97)	15.24 (15.36)	< 0.01	15.24 (16.38)	13.63 (15.26)	< 0.01	13 (15.57)	12.18 (14.39)	0.008	10.39 (12.03)	10.61 (13.08)	0.384
In-hospital mortality, n (%)^b^	213 (5.53)	304 (7.89)	< 0.01	255 (6.34)	339 (8.42)	< 0.01	261 (6.04)	311 (7.19)	0.025	249 (5.47)	307 (6.75)	0.010

*CCI* charlson comorbidity index *CHF* congestive heart failure, *HD/DP* hemodyalisis/peritoneal dialysis, *LHS* length of hospital stay

^a^Significant time trend among T2DM sufferers

^b^Significant time trend among non-T2DM sufferers

When we compared the mean CCI between those with and without T2DM we found higher values among T2DM patients for all the years analyzed.

The prevalence of risk factors such as obesity, hypertension and lipid metabolism disorders were higher among diabetic patients in the 4 years analyzed and increased significantly overtime in both groups. Furthermore, the proportion of T2DM patients hospitalized with an acute lower limb ischemia who suffered concomitant renal disease was higher than for non-diabetic patients and increased significantly overtime (9.24% in 2002 vs. 19.13% in 2014).

Regarding the use of therapeutic procedures no differences were found in the rate of major amputations between T2DM and non-diabetic patients. However a relevant and significant increment was observed in the rate of minor amputation in the T2DM group.

As can been seen in Table 5, major amputations and the use of lower extremity revascularization procedures decreased significantly over time in both groups. We found an increased rate of lower extremity angioplasty over the study period in both groups.

LHS was longer among T2DM than among non-diabetic patients, except in 2014, and decreased significantly from 2002 to 2014 in both groups.

The IHM was significantly lower in T2DM patients than in those without diabetes. In people with T2DM IHM did not change significantly over time, with figures around 5–6%.

### All cardiovascular events combined admissions

Table 6 shows the characteristics, risk factors, and diagnostic and therapeutic procedures for “all cardiovascular events combined” admissions in T2DM versus non-diabetic patients, according to the year of study. The number of T2DM patients and matched controls rose from 27,255 in 2002 to 35,406 in 2014. The proportion of men rose from 61.19% in 2002 to 63.71% in 2014 (p < 0.05) and the mean age increased from 72.03 to 73.19 years (p < 0.05).

**Table 6:** Characteristics, risk factors, and diagnostic and therapeutic procedures for all cardiovascular events combined (acute coronary syndrome, stroke, aortic aneurysm and dissection or acute lower limb ischemia) admissions in type 2 diabetes mellitus (T2DM) versus non-diabetic patients, according to year of study (2002, 2006, 2010, and 2014)

	2002			2006			2010			2014		
	T2DM	No T2DM	p value	T2DM	No T2DM	p value	T2DM	No T2DM	p value	T2DM	No T2DM	p value
N (%)^a,b^	27,255 (100)	27,255 (100)	NA	32,252 (100)	32,252 (100)	NA	35,098 (100)	35,098 (100)	NA	35,406 (100)	35,406 (100)	NA
Age adjusted rate per 100.000 inhabitants^a^	142.1	NA	NA	153.6	NA	NA	153.91	NA	NA	145.13	NA	NA
Male sex, n (%)^a,b^	16,677 (61.19)	16,677 (61.19)	NA	20,265 (62.83)	20,265 (62.83)	NA	22,168 (63.16)	22,168 (63.16)	NA	22,558 (63.71)	22,558 (63.71)	NA
Age, mean (SD)^a,b^	72.03 (10.35)	72.03 (10.35)	NA	72.56 (10.95)	72.56 (10.95)	NA	72.93 (11.44)	72.93 (11.44)	NA	73.19 (11.8)	73.19 (11.8)	NA
40–59 years, n (%)	3616 (13.27)	3616 (13.27)	NA	4503 (13.96)	4503 (13.96)	NA	5137 (14.64)	5137 (14.64)	NA	5381 (15.2)	5381 (15.2)	NA
60–69 years, n (%)	6270 (23)	6270 (23)		6607 (20.49)	6607 (20.49)		7153 (20.38)	7153 (20.38)		7359 (20.78)	7359 (20.78)	
70–79 years, n (%)	10,649 (39.07)	10,649 (39.07)		11,738 (36.39)	11,738 (36.39)		11,328 (32.28)	11,328 (32.28)		10,022 (28.31)	10,022 (28.31)	
≥ 80 years, n (%)	6720 (24.66)	6720 (24.66)		9404 (29.16)	9404 (29.16)		11,480 (32.71)	11,480 (32.71)		12,644 (35.71)	12,644 (35.71)	
CCI, mean (SD)^a,b^	2.56 (0.79)	1.49 (0.74)	< 0.01	2.64 (0.84)	1.54 (0.79)	< 0.01	2.68 (0.86)	1.58 (0.82)	< 0.01	2.79 (0.9)	1.69 (0.86)	< 0.01
Smoking, n (%)^a,b^	5393 (19.79)	6014 (22.07)	< 0.01	8101 (25.12)	9129 (28.31)	< 0.01	9768 (27.83)	10,894 (31.04)	< 0.01	10,480 (29.6)	11,857 (33.49)	< 0.01
Obesity, n (%)^a,b^	1737 (6.37)	964 (3.54)	< 0.01	2852 (8.84)	1552 (4.81)	< 0.01	3933 (11.21)	2157 (6.15)	< 0.01	4651 (13.14)	2551 (7.2)	< 0.01
Hypertension, n (%)^a,b^	14,449 (53.01)	11,428 (41.93)	< 0.01	20,005 (62.03)	15,844 (49.13)	< 0.01	22,471 (64.02)	17,963 (51.18)	< 0.01	22,523 (63.61)	18,344 (51.81)	< 0.01
Lipid metabolism disorders, n (%)^a,b^	2574 (9.44)	1740 (6.38)	< 0.01	6176 (19.15)	4238 (13.14)	< 0.01	11,461 (32.65)	8012 (22.83)	< 0.01	14,818 (41.85)	10,010 (28.27)	< 0.01
Renal diseases, n (%)^a,b^	1872 (6.87)	1272 (4.67)	< 0.01	2924 (9.07)	1899 (5.89)	< 0.01	4250 (12.11)	2638 (7.52)	< 0.01	5284 (14.92)	3427 (9.68)	< 0.01
Atrial fibrillation, n (%)^a,b^	4002 (14.68)	4464 (16.38)	< 0.01	5483 (17)	5749 (17.83)	0.003	6707 (19.11)	6958 (19.82)	0.009	7426 (20.97)	7588 (21.43)	0.098
CHF, n (%)	3605 (13.23)	2985 (10.95)	< 0.01	4347 (13.48)	3323 (10.3)	< 0.01	4792 (13.65)	3763 (10.72)	< 0.01	4871 (13.76)	3759 (10.62)	< 0.01
Mechanical ventilation, n (%)	872 (3.2)	923 (3.39)	0.209	1014 (3.14)	1061 (3.29)	0.281	1025 (2.92)	1227 (3.5)	< 0.01	1105 (3.12)	1270 (3.59)	< 0.01
HD/DP, n (%)^a,b^	181 (0.66)	140 (0.51)	0.022	286 (0.89)	190 (0.59)	< 0.01	328 (0.93)	244 (0.70)	< 0.01	399 (1.13)	249 (0.70)	< 0.01
CABG, n (%)^b^	222 (0.81)	222 (0.81)	0.905	229 (0.71)	213 (0.66)	0.439	249 (0.71)	241 (0.69)	0.714	242 (0.68)	214 (0.6)	0.188
PCI, n (%)^a,b^	2046 (7.51)	2361 (8.66)	< 0.01	4184 (12.97)	4597 (14.25)	< 0.01	5715 (16.28)	6118 (17.43)	< 0.01	6679 (18.86)	7130 (20.14)	< 0.01
Fibrinolysis, n (%)^a,b^	1659 (6.09)	1762 (6.46)	0.046	1432 (4.44)	1727 (5.35)	< 0.01	1554 (4.43)	2027 (5.78)	< 0.01	1350 (3.81)	1869 (5.28)	< 0.01
Decompressive craniectomy, n (%)^a,b^	32 (0.12)	32 (0.12)	0.603	29 (0.09)	37 (0.11)	0.326	47 (0.13)	71 (0.2)	0.028	56 (0.16)	84 (0.24)	0.017
Carotid endarterectomy, n (%)^a,b^	38 (0.14)	47 (0.17)	0.318	37 (0.11)	59 (0.18)	0.026	94 (0.27)	77 (0.22)	0.184	93 (0.26)	83 (0.23)	0.446
Carotid angioplasty, n (%)^a,b^	0 (0)	0 (0)	NA	31 (0.1)	26 (0.08)	0.501	68 (0.19)	105 (0.3)	0.005	129 (0.36)	154 (0.43)	0.131
Thoracic aorta aneurysm open surgery, n (%)^a,b^	9 (0.03)	9 (0.03)	0.913	23 (0.07)	33 (0.1)	0.127	25 (0.07)	50 (0.14)	0.001	45 (0.13)	51 (0.14)	0.460
TEVAR, n (%)^b^	0 (0)	0 (0)	NA	0 (0)	0 (0)	NA	25 (0.07)	16 (0.05)	0.133	37 (0.1)	33 (0.09)	0.600
Abdominal aorta aneurysm open surgery, n (%)	87 (0.32)	96 (0.35)	0.406	112 (0.35)	114 (0.35)	0.881	100 (0.28)	135 (0.38)	0.011	137 (0.39)	156 (0.44)	0.204
EVAR, n (%)^a,b^	20 (0.07)	22 (0.08)	0.758	95 (0.29)	78 (0.24)	0.145	234 (0.67)	194 (0.55)	0.019	314 (0.89)	293 (0.83)	0.253
Minor amputation, n (%)^a,b^	585 (2.15)	356 (1.31)	< 0.01	626 (1.94)	333 (1.03)	< 0.01	646 (1.84)	345 (0.98)	< 0.01	652 (1.84)	383 (1.08)	< 0.01
Major amputation, n (%)^a,b^	594 (2.18)	623 (2.29)	0.337	624 (1.93)	599 (1.86)	0.396	568 (1.62)	544 (1.55)	0.416	500 (1.41)	538 (1.52)	0.183
Lower extremity revascularization, n (%)^a,b^	1016 (3.73)	950 (3.49)	0.076	1064 (3.3)	1014 (3.14)	0.209	952 (2.71)	1032 (2.94)	0.038	852 (2.41)	990 (2.8)	< 0.01
Lower extremity angioplasty, n (%)^a,b^	378 (1.39)	351 (1.29)	0.289	624 (1.93)	507 (1.57)	< 0.01	1077 (3.07)	840 (2.39)	< 0.01	1456 (4.11)	1144 (3.23)	< 0.01
LHS, mean (SD)^a,b^	12.41 (13.28)	11.87 (13.65)	< 0.01	11.51 (12.92)	10.87 (13.21)	< 0.01	10.44 (13.22)	10.22 (14.28)	0.030	9.1 (10.52)	9.08 (11.11)	0.837
In-hospital mortality, n (%)^a,b^	3555 (13.04)	3464 (12.71)	0.226	4002 (12.41)	3909 (12.12)	0.242	4134 (11.78)	4066 (11.58)	0.402	3814 (10.77)	3728 (10.53)	0.269

*CCI* charlson comorbidity index, *CHF* congestive heart failure, *HD/DP* hemodyalisis/peritoneal dialysis, *CABG* coronary artery bypass graft, *PCI* percutaneous coronary intervention, *TEVAR* endovascular implantation of graft in thoracic aorta, *EVAR* endovascular implantation of graft in abdominal aorta, *LHS* length of hospital stay

^a^Significant time trend among T2DM sufferers

^b^Significant time trend among non-T2DM sufferers

For all the years analyzed, higher values of hemodyalisis and peritoneal dialysis were found in T2DM patients for “all cardiovascular events combined” admission and increased significantly in both group overtime.

LHS decreased significantly over time both for T2DM and non-diabetic people. The IHM for the T2DM population was 13.04% in 2002 and 10.77% in 2014 (p < 0.05). Equivalent figures for the non-diabetic population were significantly lower, 12.71 and 10.53% respectively.

**Fig. 1:**
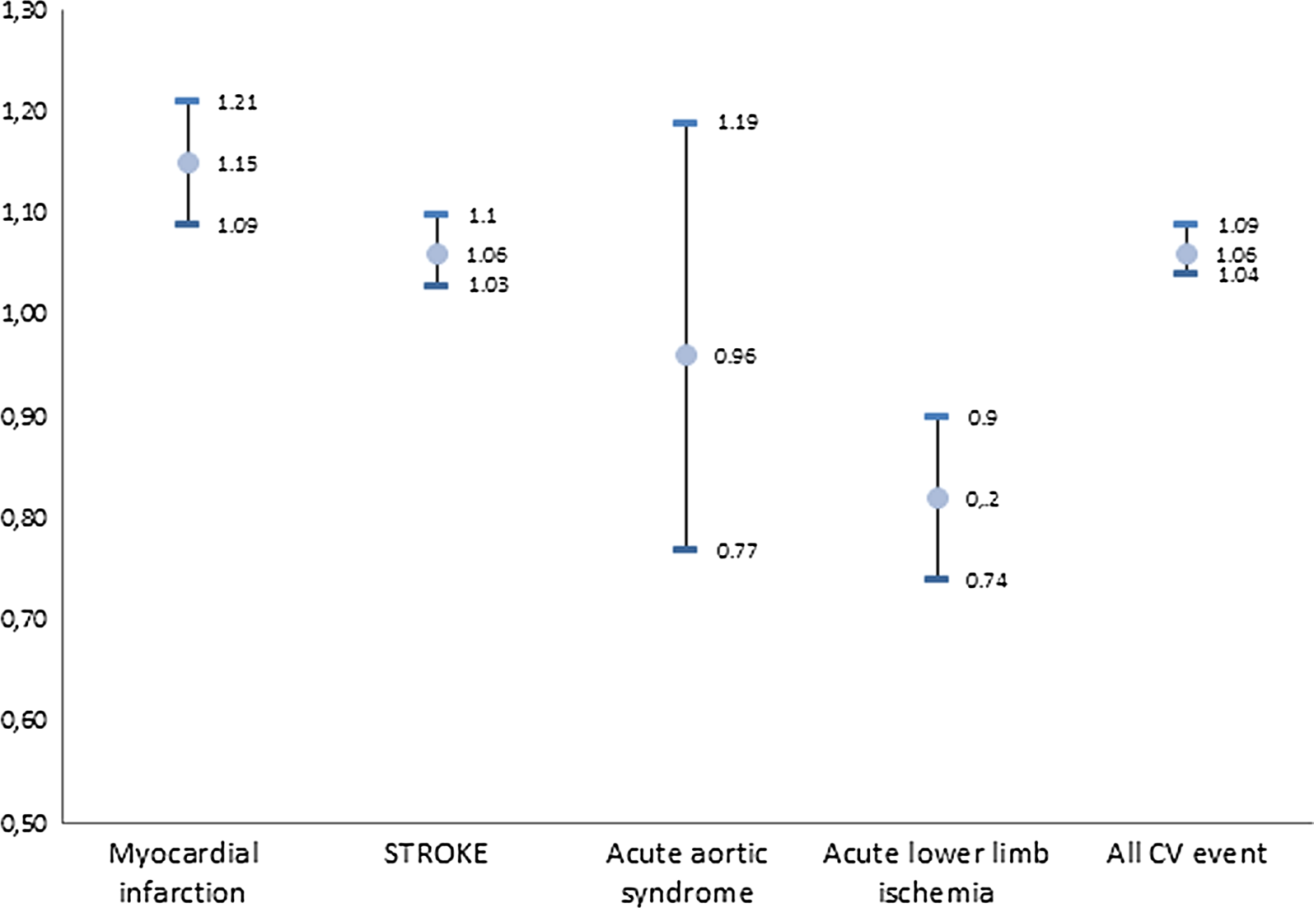
Conditional logistic regression analysis to assess the effect of T2DM on the IHM in patients with acute cardiovascular events admissions. Odds ratios (ORs) with their 95% confidence intervals (CIs)

Figure 1 shows the results of the conditional logistic regression analysis to assess the effect of T2DM on the IHM in patients with acute cardiovascular events admissions. After adjusting for possible confounders T2DM patients had significantly higher mortality rates during admission for myocardial infarction (OR 1.15; 95% CI 1.09–1.21) and stroke (OR 1.06; 95% CI 1.03–1.10). However these patients had a significantly lower mortality when admitted for acute lower limb ischemia (OR 0.82; 95% CI 0.74–0.90).

Finally, we found that T2DM patients had a 6% higher probability (OR 1.06; 95% CI 1.04–1.09) of dying during the hospitalization for “all cardiovascular events combined” than non-diabetic matched controls.

## Discussion

In the Spanish population, we found increasing numbers of admissions overtime for stroke, aortic aneurysm and dissection and acute lower limb ischemia, but not for myocardial infarction, which remained stable. Previous reports had highlighted declining incidence rates for myocardial infarction in western societies, perhaps as a consequence of the preventive measures implemented to lower cardiovascular risk [4]. Notwithstanding the previous statement, people admitted to hospitals in our country for major cardiovascular events were progressively older (except for myocardial infarction), had more comorbidities (especially people with T2DM), and were more frequently coded overtime for classical cardiovascular risk factors (smoking, obesity, hypertension, lipid disorders) and renal diseases. This particularity was seen in people both with and without T2DM, albeit these factors were more prevalent in the T2DM population, except for smoking. However, a better quality coding in recent years could not be ruled out as a potential confounder [11]. Also, despite the lowering incidence rates of stroke previously reported by others [12], more recent research [13], including our own previous reports [6] support increasing numbers of admissions for this condition.

Atrial fibrillation was more often coded in the non-diabetic population and was increasingly coded overtime in people with and without T2DM. Contrarily, congestive heart failure was more often coded in people with T2DM, and remained stable overtime in both populations. This stability had been underscored in recent research [14], and some authors claim that new drugs with course-modifying properties may be playing a role to avoid readmissions [15]. In follow up studies it has been found, that among patients with heart failure both all-cause and cardiovascular mortality were higher in diabetic patients [16]. However, in Spain a study showed that in hospital mortality in patients admitted because of acute heart failure did not differ between those with and without type 2 diabetes [17].

PCI increased overtime and was more frequently done in people without diabetes, at the expense of lowering rates of fibrinolysis during admission for myocardial infarction. Updated guidelines support prompt PCI where available, with a performance goal of ≤ 90 min from the first medical contact for ST elevation myocardial infarction within the previous 12 h [18].

The use of Coronary Artery Bypass Graft among diabetic patients remained stable overtime in our investigation. A recent meta-analysis concluded that CABG was associated with a significantly lower overall mortality rate and with less cerebrovascular and major adverse cardiovascular events than PCI among insulin-treated type 2 diabetes mellitus patients [19].

As opposed to myocardial infarction, fibrinolysis was more frequently performed overtime during admission for an ischemic stroke, with predominance in people without diabetes. We had previously reported a higher mortality rate in T2DM people admitted for ischemic stroke undergoing systemic fibrinolysis [6], which might be due to a higher risk of bleeding in T2DM patients after systemic fibrinolytic therapy [20]. We do not know whether the perception of this risk could be influencing the decision to withhold fibrinolysis in the T2DM population; alternatively, particular characteristics of ischemic stroke in people with diabetes might make physicians less prone to indicate this treatment.

Major lower limb amputations declined overtime, with a more widespread use of lower limb angioplasty. A combined approach of improving regional blood flow plus conservative minor amputations is probably driving the declining rate of major amputations, as pointed out too by previous research [21]. This is relevant because lower-extremity amputations and the history of peripheral revascularization have been associated with increased risk of all-cause mortality and major macrovascular events [22].

LHS was lower in people without diabetes overall, but it declined in both populations overtime. For myocardial infarction our result agree with Loudon et al., who described for acute coronary syndromes a mean LHS of 7.8 days for T2DM patients compared with 7 days for the entire cohort (Adjusted OR 1.003; 95% CI 1.001–1.004) [23].

IHM declined overtime, though this trend was not significant for people without diabetes in the last 2 years analyzed. Other authors had described progressively lower mortality rates for cardiovascular mortality in Europe during the last three decades [24], yet with some heterogeneity within its borders [25, 26]. Patients’ awareness of classical cardiovascular risk factors, global preventive measures, free access to all levels of health care and prompt invasive management of acute conditions have probably acted synergistically to improved outcomes of cardiovascular disease [27].

In the multivariate analyses, T2DM patients had a significantly 15% higher mortality rate during admission for myocardial infarction, a 6% higher mortality for stroke and a 6% higher mortality for “all cardiovascular events combined”, than non-diabetic matched controls. Myocardial infarction has long been associated with a higher short and long-term mortality in people with T2DM [28–31]. Lettino et al. using 10 contemporary registries in Europe compared outcomes for acute coronary syndromes (ACS) between patients with and without diabetes mellitus. Their results showed a 1.66 (95% CI 1.42–1.94) higher risk of in-hospital all-cause death among diabetic patients [30]. Using the National Inpatient Sample, Ahmed et al., obtained an adjusted OR of 1.069, (95% CI 1.051–1.087) for DM on in-hospital mortality in patients with an acute myocardial infarction [31]. It is reassuring that in previous research from our group, without proceeding with case–control matching and evaluating a different period of time (2001–2010), the IHM that we reported was 14% higher in people with T2DM compared with no-diabetes in people admitted for a myocardial infarction [5], which is very similar to current data. Also, we had formerly reported a 7% significantly higher mortality for women with T2DM admitted for ischemic stroke versus non-diabetic women, yet no statistical differences for men [6], or for any gender when comparing T2DM and non-diabetic patients admitted for hemorrhagic stroke [32]. The differences in survival after a stroke between people with and without T2DM seem to persist in the long-term: Eriksson et al. showed that during a follow-up time after a stroke of 86,086 patient-years, 75.7% of the diabetic patients and 58.5% of the non-diabetic patients had died (p < 0.001), with median survivals of 60 months (95% CI 57–64) for the former and 117 months (95% CI 113–120) for the latter [33].

The prevention of cardiovascular events is of great importance for long-term survival of T2DM patients. Necessary strategies include intensive glycemic and risk factors control, an appropriate selection of glucose-lowering medications and screening strategies for the early detection of cardiovascular complications [34–37]. The benefit of aggressive coronary screening must be considered [37].

The robustness of our findings is supported by the large sample size, the 12-year follow-up period virtually covering all the Spanish population and the standardized methodology, which has been previously used for research in diabetes and its complications [38]. We were able to select matched controls for the T2DM cases to lower the possibility of confounding due to different baseline characteristics between the two subpopulations. Nevertheless, our work has some limitations: Our data source was the CMBD, an administrative database that contains discharge data for hospitalizations in Spain and depends on the quality of the information that physicians report.

The prevalence of obesity, hypertension and smoking (including prior smoking) found in our investigation are lower than those reported in other studies conducted among people with diabetes in Spain [39–41].

The under-coding of obesity is relevant since, previous investigations have suggested that among diabetic patients obesity may have a protective effect on cardiac and all-cause mortality after adjusting for confounding factors [42].

Previous studies have found that under reporting of risk factors including obesity is common among discharge diagnoses [43, 44].

The main reason for this is that according to the SNHDD methodology the primary/main diagnosis is defined as the condition which, after proper investigation, is considered the reason why the patient was admitted to the hospital [9]. The secondary diagnosis includes those diseases or risk factors that coexists with the primary diagnosis at the time of admission or were detected during the hospitalization and that, in the opinion of the treating physician, may have affected the patient’s progress or treatment plan. Other possible reasons include that people who codify may not record risk factors owing to time constraints when performing data abstraction or that when time for coding is limited, coders tend to include severe conditions but not risk factors [43, 44].

Another limitation is that coding of diagnoses and guidelines of reporting may have substantially changed over the study period. However, in Spain the SNHDD has maintained the same methodology over the last years and the codes for the cardiovascular conditions studied and diabetes have not been modified overtime. In any case these changes in coding would not affect the main results of our investigation because we compare always T2DM patients with matched non diabetic patients who were hospitalized the same year, so it is expected that the changes in reporting would affect equally cases and controls. However, these changes in diagnostic criteria, coding practices and reporting could affect time trend analysis. Beside these limitations studies conducted in other countries have assessed the time trends in hospitalizations for cardiovascular diseases using hospital discharge data [45, 46].

Unfortunately in Spain a validation study to assess the rate of unreported diagnosis of diabetes in administrative databases has not yet been conducted, to our knowledge. However, Leong et al. reported that a commonly-used administrative database definition for diabetes had a sensitivity of 82.3% (95% CI 75.8, 87.4) and a specificity of 97.9% (95% CI 96.5, 98.8%) [47]. While this definition misses about one-fifth of cases of diabetes and wrongly classifies 2.1% of non-cases in the population as diabetes cases, it is likely sensitive enough for analyzing trends in the general population, if its accuracy remains reasonably stable overtime.

We used four prespecified years (2002, 2006, 2010, 2014) as representative of the time trends in number of cases and outcomes of the admissions for cardiovascular events in our population, thus lacking continuous information for the whole 12-year period. Our database is also limited by its anonymity (no identifying items, such as clinical history number), what precludes the extraction of some specific pieces of information (i.e., people who moved from one hospital to another would appear twice). We had no access to additional variables, such as duration of diabetes or drugs exposure.

Finally, a possible methodological limitation is that we did not match for the comorbidities included in the CCI. According to several authors matching for too many variables may lead to overmatching that would not only decrease the number of case–control pairs but also probably increase the number of concordant (uninformative) pairs [48–50]. In fact, in our study matching for the CCI would reduce the number of matched pair to under 40% of diabetic patients.

Szklo concludes that over-adjustment occurs when adjustment (or matching) is carried out for a variable so closely related to the variable of interest that no variability in the latter is allowed. For example in case–control study, making the case and control groups very similar o identical regarding the confounder results in their also very similar or identical regarding the exposure, thereby resulting in apparent null association. In general it must be kept in mind that when adjustment is carried out for a given confounding variable, it is also carried out for variables related to it [48].

So, to avoid losing sample size and overmatching we considered a better option to match for only three variables, age, sex and province of residence including comorbidity using the CCI as a covariable within the multivariable model, since it allowed us to explore its role over the outcomes, avoiding the risk of overmatching and maximizing the number of informative pairs.

## Conclusions

In Spain, the number of hospital admissions for stroke, aortic aneurysm and dissection and acute lower limb ischemia increased overtime (2002–2012), but remained stable for myocardial infarction. LHS and IHM declined overtime, though IHM only did it significantly in all years analyzed in people with T2DM. T2DM patients had a significantly 15% higher mortality rate during admission for myocardial infarction, a 6% higher mortality for stroke, and a 6% higher mortality rate for “all cardiovascular events combined”, than non-diabetic matched controls. Further research is needed to confirm these findings in countries similar to ours, and to help us understand the reasons for an apparently increased mortality in T2DM patients when admitted to hospital for some major cardiovascular events.

## Abbreviations

CIs: confidence intervals
SNHDD: Spanish National Hospital Discharge Database
EVAR: endovascular implantation of graft in abdominal aorta
ICD-9-CM: International Classification of Diseases, Ninth Revision, Clinical Modification
IHM: in-hospital mortality
LHS: length of hospital stay
MHSSE: Spanish Ministry of Health, Social Services and Equality
ORs: odds ratios
PCI: percutaneous coronary intervention
T2DM: type 2 diabetes mellitus
TEVAR: endovascular implantation of graft in thoracic aorta
